# Trauma exposure and factors associated with ICD-11 PTSD and complex PTSD in adolescence: a cross-cultural study in Japan and Lithuania

**DOI:** 10.1017/S2045796022000336

**Published:** 2022-07-11

**Authors:** E. Kazlauskas, L. Jovarauskaite, K. Abe, C. R. Brewin, M. Cloitre, I. Daniunaite, Y. Haramaki, S. Hihara, A. Kairyte, Y. Kamite, K. Sugimura, S. Thoresen, P. Zelviene, I. Truskauskaite-Kuneviciene

**Affiliations:** 1 Center for Psychotraumatology, Institute of Psychology, Vilnius University, Vilnius, Lithuania; 2 Graduate School of Humanities and Social Sciences, Hiroshima University, Hiroshima, Japan; 3 Research Department of Clinical, Educational and Health Psychology, University College London, London, UK; 4 National Center for PTSD Dissemination and Training Division, Veterans Affairs Palo Alto Health Care System, Palo Alto, CA, USA; 5 Department of Psychiatry and Behavioral Sciences, Stanford University, CA, USA; 6 Norwegian Center for Violence and Traumatic Stress Studies, Oslo, Norway

**Keywords:** Adolescence, Japan, Lithuania, posttraumatic stress disorder, stressful life events, trauma

## Abstract

**Aims:**

Cross-cultural studies of posttraumatic stress disorder (PTSD) and complex PTSD (CPTSD) based on ICD-11 diagnostic criteria are scarce, especially in adolescence. The study aimed to evaluate the trauma exposure, prevalence and factors associated with PTSD and CPTSD in general populations of adolescents in Lithuania and Japan.

**Methods:**

The study sample comprised 1746 adolescents from Lithuania (*n* = 832) and Japan (*n* = 914), 49.8% female. The mean age of study participants was 15.52 (s.d. = 1.64), ranging from 12 to 18 years. ICD-11 posttraumatic disorders were assessed using the International Trauma Questionnaire – Child and Adolescent version (ITQ-CA).

**Results:**

More than half of the adolescents in a total sample (61.5%) reported exposure to at least one traumatic event in their lifetime, 80.0% in Lithuania and 44.6% in Japan, with a higher prevalence of interpersonal trauma in Lithuania and more natural disaster exposure in Japan. The prevalence of PTSD was 5.2% (95% CI 3.8–6.9%) and 2.3% (95% CI 1.4–3.5%), CPTSD 12.3% (95% CI 10.1–14.7%) and 4.1% (95% CI 2.9–5.5%) in Lithuanian and Japanese samples, respectively. Cumulative trauma exposure, female gender, loneliness and financial difficulties in family predicted both PTSD and CPTSD in the total sample. Loneliness discriminated CPTSD *v*. PTSD in both Lithuanian and Japanese samples.

**Conclusions:**

This cross-cultural study is among the first which reported different patterns of trauma exposure in Asian Japanese and Lithuanian adolescents in Europe. Despite differences in trauma exposure and PTSD/CPTSD prevalence, we found similar predictors in both studies, particularly the importance of cumulative trauma exposure for PTSD/CPTSD, and social interpersonal factors for the risk of CPTSD. The study supports the universality of traumatic stress reactions to adverse life experiences in adolescence across cultures and regions and highlights different levels of traumatisation of adolescents in various countries.

## Introduction

Traumatic experiences in childhood and adolescence significantly impact mental health and may lead to disrupted psychosocial development (Lewis *et al*., 2019). Adolescents are vulnerable to mental disorders, especially if exposed to interpersonal violence, such as physical or sexual abuse (Silva *et al*., 2020). Unfortunately, adolescents are a vastly understudied population in the traumatic stress literature. Most posttraumatic stress disorder (PTSD) studies are conducted in adult populations, such as military veterans, or following natural disasters, terror attacks or other adversities. Furthermore, PTSD studies in adolescence are predominantly conducted in high-income North American or European countries, such as Norway or the USA. The impact of traumatic experiences on adolescents in other regions across the globe, such as Asia or Africa, is still relatively unknown.

The recent inclusion of complex PTSD (CPTSD) in the 11th revision of the International Classification of Diseases (ICD-11) by the World Health Organization (WHO) (World Health Organization, 2018) fostered a worldwide interest in CPTSD, and studies of CPTSD measures development, prevalence or risk factors. However, most such studies conducted in adult populations (e.g. Brewin *et al*., 2017; Kvedaraite *et al*., 2021; Redican *et al*., 2021) did not provide enough evidence on CPTSD in adolescence. The development of the self-report measure for children and adolescents, a modified version of the widely used International Trauma Questionnaire (ITQ) initially developed for an adult population (Cloitre *et al*., 2018), only recently provided the first insights into CPTSD in adolescence (Haselgruber *et al*., 2020a; Kazlauskas *et al*., 2020). A growing number of studies confirm that the ICD-11 symptom structure in adolescents is similar to that of adult PTSD and CPTSD (Sachser *et al*., 2017b; Haselgruber *et al*., 2020b; Kazlauskas *et al*., 2020). Thus, empirical evidence so far supports that the same diagnostic criteria could be applied in identifying CPTSD in both adult and adolescent populations. Furthermore, the first studies on the CPTSD prevalence and risk factors (Elliott *et al*., 2021; Haselgruber *et al*., 2021; Tian *et al*., 2021; Redican *et al*., 2022) identified discriminating factors of CPTSD *v*. PTSD, with the social factors, such as lack of social support or difficulties in family or school, having a specifically important role (Daniunaite *et al*., 2021).

Despite several attempts to have global estimations of trauma exposure and PTSD prevalence (e.g. Kessler *et al*., 2017), most studies worldwide report the prevalence of trauma and PTSD using various methods. Furthermore, data are often collected at different time points, which might hugely affect the outcomes of such studies in rapidly changing societies around the globe affected by such challenges as climate change-related disasters, pandemics or political instability. In the ICD-11 CPTSD research, the ITQ is currently a widely used self-report measure. Its use enables either merging the CPTSD datasets from different countries (e.g. Knefel *et al*., 2020) or comparing empirical studies in systematic reviews (e.g. Brewin *et al*., 2017; Redican *et al*., 2021). However, CPTSD studies that use the harmonised methodology are critical as such studies would provide more rigid comparisons of the prevalence of PTSD/CPTSD and risk factors, in particular, in a novel area of adolescent complex trauma (Cloitre *et al*., 2009).

The current study aimed to estimate the prevalence of traumatic experiences, PTSD and CPTSD based on ICD-11 criteria and factors associated with PTSD and CPTSD in Lithuania and Japan. The two countries from Europe and Asia included in the study represent different cultural contexts. Lithuania has a history of rapidly changing political and social situations over the last several decades after the collapse of the Soviet Union in the early 1990s (Kazlauskas and Zelviene, 2016). Japan is an Asian country with a collectivistic culture and a technologically developed population with a complex history and high exposure to natural disasters. We included social factors, such as social support, loneliness, financial difficulties in family along with trauma exposure in the study to explore factors associated with PTSD/CPTSD in adolescence based on previous empirical evidence (Daniunaite *et al*., 2021) as relevant to the cultural contexts in both countries in our study.

## Methods

### Participants and procedures

This study is part of the larger multicultural longitudinal study *Stress and Resilience in Adolescence* (STAR-A) initiated at the Center for Psychotraumatology at Vilnius University in Lithuania (Kazlauskas *et al*., 2020; Zelviene *et al*., 2020; Daniunaite *et al*., 2021). The relevant Institutional Review Boards approved the study in both Japan and Lithuania. Informed parental or official guardian consent, additionally ascend for participation from the adolescents in both countries have been obtained before the data collection. Cross-sectional data from the third wave of the Lithuania STAR-A study and the first wave of the Japan STAR-A study were included in the analysis to match the data collection timeframe in the two countries.

Data collection in educational settings across various regions of Lithuania took place in March–June 2021. Due to the COVID-19 pandemic restrictions and lockdown in Lithuania, data were collected using the platform designed for online surveys. Each school was contacted, and the time for the data collection was set. The researcher or trained and supervised student researcher participated in online adolescent data collection meetings to explain the procedures and answer study participants' questions. We reached out to 1299 adolescents from 49 schools, and 856 adolescents filled in an online survey with a response rate of 65.9%. The response rate was affected by the temporary closure of schools due to COVID-19 pandemic-related restrictions. Responses of 22 adolescents (2.6%) in the Lithuanian sample were excluded due to insufficient data for data analysis.

Data in Japan were collected in June–July 2021. Adolescents from various regions of Japan responded to the online survey through the crowdsourcing website Lancers (https://www.lancers.jp), which has one of the highest numbers of registrants in Japan. Adolescents of age ⩽18 years are not allowed to register on this website by themselves; hence, registrants who have child(ren) in junior high school or high school received notification of the call for survey participation, information about the procedure of this study and a hyperlink to the online survey. Participants in Japan received small financial compensation for participation in the survey of 1000 JPY (about 7.6 EUR); no financial incentives were offered for study participants in Lithuania. For the Japanese sample, the response rate was not available as the recruitment was via a large panel of participants of the survey company. In total, 918 adolescents filled in the survey in the Japanese sample, and responses of four (0.4%) participants were excluded due to insufficient data.

The final study sample comprised 1746 adolescents, Lithuania (*n* = 832) and Japan (*n* = 914), 49.8% female; age *M* (s.d.) = 15.52 (1.64); range 12–18. The participants' characteristics in the total sample and each country are presented in Table 1. As we included participants in a highly diverse cultural context, we aimed to recruit participants of similar age adolescents from the general population in both countries using a harmonised methodology and same time in data collection. However, we did not expect samples to match all the sociodemographic characteristics due to the specific cultural contexts in both countries. The final sample was comparable but differed in its sociodemographic characteristics between the two country samples (see Table 1).

**Table 1.** Demographic characteristics of participants (*N* = 1746)

Demographic characteristic	Total sample	Country		Significance statistics
		Japan *n* *=* 914	Lithuania *n* = 832	
	*N* (%)	*n* (%)	*n* (%)	
Age *M* (s.d.)	15.52 (1.64)	14.93 (1.67)	16.17 (1.34)	*t*(1716.90) *=* 17.22, *p* < 0.001
Range	12–18	12–18	13–18	
Gender				*χ*^2^(1) = 56.49, *p* < 0.001
Female	870 (49.8)	377 (41.2)	493 (59.3)	
Male	876 (50.2)	537 (58.8)	339 (40.7)	
Living with				*χ*^2^(2) = 103.78, *p* < 0.001
Both parents/foster parents	1422 (81.4)	826 (90.4)	596 (71.6)	
Single parent	298 (17.1)	77 (8.4)	221 (26.6)	
Other (e.g. relatives, institution)	26 (1.5)	11(1.2)	15 (1.8)	
Mother employed				*χ*^2^(2) = 231.31, *p* < 0.001
Yes	1388 (79.5)	657 (71.9)	731 (87.9)	
No	106 (6.1)	21 (2.3)	85 (10.2)	
Do not know	252 (14.4)	236 (25.8)	16 (1.9)	
Father employed				*χ*^2^(2) = 50.77, *p* < 0.001
Yes	1583 (90.7)	869 (95.1)	714 (85.8)	
No	65 (3.7)	26 (2.8)	39 (4.7)	
Do not know	98 (5.6)	19 (2.1)	79 (9.5)	
Financial difficulties in family				*χ*^2^(1) = 102.40, *p* < 0.001
No	1597 (91.5)	777 (85.0)	820 (98.6)	
Yes	149 (8.5)	137 (15.0)	12 (1.4)	
Received professional support of mental health professional over last 12 months				*χ*^2^(2) = 42.79, *p* < 0.001
None	1605 (91.9)	876 (95.8)	729 (87.6)	
Yes, one or several sessions	97 (5.6)	31 (3.4)	66 (7.9)	
Yes, at least several months or more	44 (2.5)	7 (0.8)	37 (4.5)	

### Measurements

#### Lifetime trauma exposure

Lifetime trauma exposure was assessed with the traumatic experiences checklist from the Child and Adolescent Trauma Screen (CATS) (Sachser *et al*., 2017a). The checklist comprises 14 potentially traumatic experiences (e.g. natural disaster, physical abuse, sexual abuse, see Table 2), with dichotomous answers *Yes/No* to each of the listed experiences. Participants were considered exposed to potentially traumatic experiences if they indicated at least a single experience in the checklist. Cumulative trauma exposure was estimated by summing all the indicated traumatic events ranging from 0 to 14.

**Table 2.** Traumatic experiences in study sample (*N* = 1746)

Traumatic experiences		Country			
	Total sample	Japan *n* = 914	Lithuania *n* = 832		
	*N* (%)	*n (*%)	*n (*%)	χ^2^ (1)	*p*
Natural disaster	242 (13.9)	160 (17.5)	82 (9.9)	21.35	<0.001
Serious accident or injury	503 (28.8)	103 (11.3)	400 (48.1)	287.71	<0.001
Robbed by threat, force or weapon	49 (2.8)	17 (1.9)	32 (3.8)	6.29	0.012
Slapped, punched or beat up in your family	260 (14.9)	108 (11.8)	152 (18.3)	14.31	<0.001
Slapped, punched or beat up by someone else	281 (16.1)	76 (8.3)	205 (24.6)	85.95	<0.001
Seeing someone in your family get slapped, punched or beat up	225 (12.9)	87 (9.5)	138 (16.6)	19.38	<0.001
Seeing someone in your community get slapped, punched	420 (24.1)	86 (9.4)	334 (40.1)	225.21	<0.001
Someone older touching your private parts when they shouldn't	63 (3.6)	24 (2.6)	39 (4.7)	5.23	0.021
Someone forcing or pressuring sex, or when you couldn't say no	47 (2.7)	8 (0.9)	39 (4.7)	24.17	<0.001
Someone close to you dying suddenly or violently	267 (15.3)	55 (6.0)	212 (25.5)	127.37	<0.001
Attacked, stabbed, shot at or hurt badly	31 (1.8)	7 (0.8)	24 (2.9)	11.21	0.001
Seeing someone attacked, stabbed, shot at, hurt badly or killed	81 (4.6)	13 (1.4)	68 (8.2)	44.87	<0.001
Stressful or scary medical procedure	347 (19.9)	26 (2.8)	321 (38.6)	349.31	<0.001
Being around war	21 (1.2)	8 (0.9)	13 (1.6)	1.73	0.188
At least one traumatic event	1074 (61.5)	408 (44.6)	666 (80.0)	230.66	<0.001
Cumulative trauma					
1	399 (22.9)	230 (25.2)	169 (20.3)	5.82	0.016
2–3	402 (23.0)	137 (15.0)	265 (31.9)	69.87	<0.001
4–5	171 (9.8)	25 (2.7)	146 (17.5)	108.17	<0.001
⩾6	102 (5.8)	18 (2.0)	86 (10.3)	58.37	<0.001
*Mean* (s.d.)	1.62 (1.99)	0.85 (1.43)	2.47 (2.17)	*t*(df) = 18.28 (1412.69)	<0.001

#### Posttraumatic stress reactions

The International Trauma Questionnaire – Child and Adolescent (ITQ-CA) version (Cloitre *et al*., 2018; Kazlauskas *et al*., 2020) was used to measure PTSD symptoms based on ICD-11 diagnostic criteria. The ITQ-CA consists of two main parts with three symptom clusters in each. The first part measures PTSD symptoms and includes reexperiencing (Re), avoidance (Av) and sense of current threat (SoT) symptoms. The second part measures disturbances in self-organisation (DSO) symptoms and includes evaluation of affective dysregulation (AD), negative self-concept (NSC) and disturbances in relationships (DR). Each symptom cluster includes two symptom items with 12 symptom items of the ITQ-CA. Participants were asked to evaluate how each symptom bothered them in the past month using a five-point Likert scale ranging from *Never* ( = 0) to *Very Often* ( = 4). Five functional impairment questions are presented in the ITQ-CA separately following PTSD and DSO symptoms items. The respondent is asked to indicate if any of PTSD or DSO symptoms interfered with persons' relationships with friends, family, school, general happiness and any other important living area, with binary answers, *No/Yes*. PTSD diagnosis is considered if at least one of two items that comprise each of the PTSD symptom clusters was rated at ⩾2, and at least one of five functional impairment items was indicated positively in association with PTSD symptoms. CPTSD diagnosis was considered if an individual met full criteria for PTSD, and at least one item in each DSO symptom cluster was endorsed with a rating of ⩾2, and at least one functional impairment item associated with DSO symptoms was indicated as positive. Translation procedures with several iterations of revisions using a back-translation procedure for Lithuanian and Japanese language versions of the ITQ-CA were used. The ITQ-CA has been used previously in Lithuania (Kazlauskas *et al*., 2020). The internal reliability of all ITQ-CA symptom items was good for both Lithuanian and Japanese versions, *α* = 0.91 and *α* = 0.91, respectively, as were the internal reliability estimates for the PTSD symptoms (Lithuanian *α* = 0.84; Japanese *α* = 0.87) and DSO symptoms (Lithuanian *α* = 0.89; Japanese *α* = 0.89).

#### Loneliness

Loneliness was assessed with the three-item loneliness scale (Hughes *et al*., 2004) comprised of items measuring how often participants feel like (1) they are missing being with other people, (2) left behind others and (3) isolated from others. Participants were asked to select responses on a three-point scale *Never* ( = 0), *Sometimes* ( = 1) and *Often* ( = 2) to each of the questions. A loneliness score was obtained by summing responses to all three items, with a higher score indicating higher loneliness. This scale has been used in a previous study of loneliness in adolescence (Thoresen *et al*., 2018). The internal reliability of the loneliness scale was good in both Lithuanian and Japanese samples (Lithuanian version *α* = 0.79; Japanese version *α* = 0.80).

#### Perceived positive social support

Perceived positive social support (PPSS) was measured by using a revised brief version of the Crisis Support Scale (Joseph *et al.*, 1992). The PPSS comprises four items measuring instrumental and emotional social support: (1) how often does someone tend to listen if the person wants to talk, (2) can the person talk about his/her thoughts and feelings with others, (3) do people sympathise and support a person and (4) does anyone help with everyday practical problems. A seven-point Likert scale ranging from *Never* ( = 1) to *Always* ( = 7) for each PPSS item evaluation was used. PPSS total score constituted the sum of the responses to all four items, with higher scores indicating receiving more perceived social support. The internal reliability of the PPSS scale was good in the Lithuanian version (*α* = 0.88) and the Japanese version (*α* = 0.88).

### Data analyses

All the participants included in the data analysis were compliant with the study procedures and filled in the online survey without missing data.

**Fig. 1.**
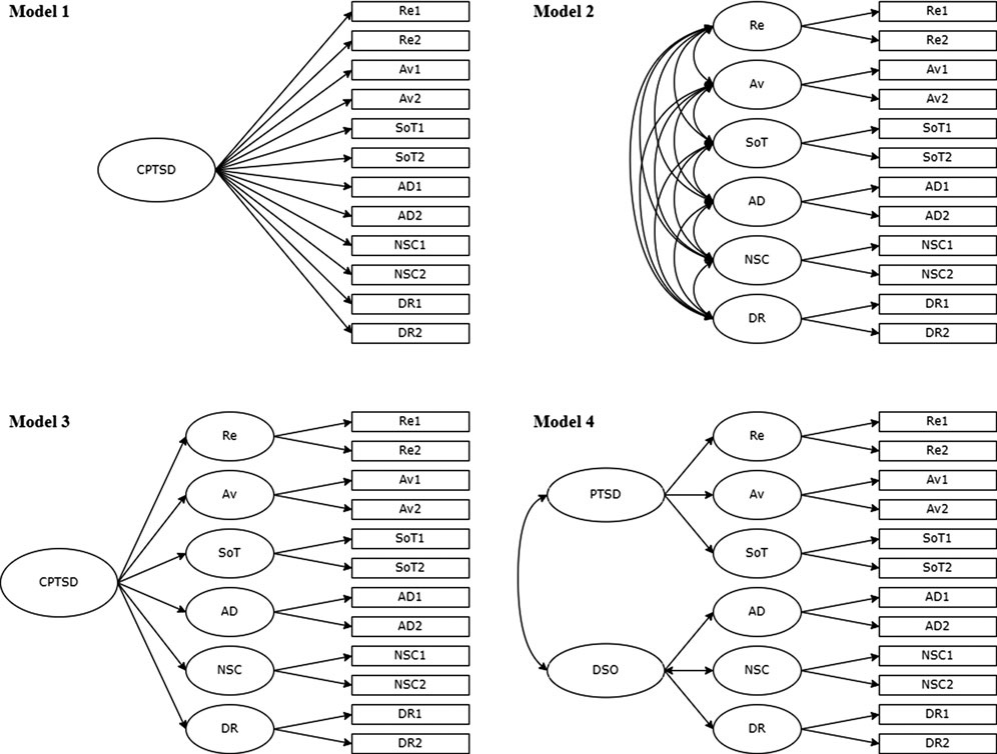
Factor models of ITQ-CA tested in the study using confirmatory factor analysis. CPTSD, complex posttraumatic stress disorder; PTSD, posttraumatic stress disorder; DSO, disturbances in self-organisation; Re, re-experiencing; Av, avoidance; SoT, sense of current threat; AD, affect dysregulation; NSC, negative self-concept; DR, disturbed relationships.

Confirmatory factor analysis (CFA) was used to estimate the factor structure of the ITQ-CA in Lithuanian and Japanese samples separately, as well as in the total sample. We tested four CFA models of PTSD and CPTSD symptom structure (see Fig. 1), that was tested in previous studies (Haselgruber *et al*., 2020a; Kazlauskas *et al*., 2020). Model 1 was a one-factor first-order model, where all 12 ITQ-CA symptom items were loaded onto a single CPTSD latent factor. Model 2 was a first-order correlated six-factor model (Re, Av, SoT, AD, NSC, DR). Model 3 was a second-order one-factor model in which one second-order factor of CPTSD explained the covariation of the six-factor model. Model 4 was a correlated second-order two-factor PTSD and DSO model, in which correlations between Re, Av and SoT were explained by the PTSD factor, and AD, NSC and DR were explained by the DSO factor. The CFA models were estimated using the Robust Maximum Likelihood (MLR) estimator. These models' fits were assessed using the *χ*^2^ test, the root-mean-square error of approximation (RMSEA), the comparative fit index (CFI), Tucker–Lewis index (TLI) and the standardised root-mean-square residual (SRMR) indices. The results of RMSEA and SRMR values of ⩽0.08, CFI and TLI values ⩾0.90, and a non-significant *χ*^2^ result indicate a good model fit (Kline, 2011).

Further, the measurement invariance test was used to check whether the ITQ-CA could be used in both Lithuanian and Japanese cultures for measuring PTSD/CPTSD. Model comparisons were conducted by examining the changes in fit indices, ΔCFI ⩽ 0.010 and ΔRMSEA ⩽ 0.015 indicate no significant differences between models (Chen, 2007).

Multinomial logistic regression was used to determine the predictors of PTSD and CPTSD in a sample of Lithuanian and Japanese adolescents who experienced at least one traumatic experience. Sociodemographic, trauma-related and social-interpersonal factors were included as predictors in all models: gender, country, socioeconomic difficulties, cumulative trauma, loneliness and PPSS. Multinomial logistic regression was conducted with no diagnosis groups and PTSD group as reference groups. Cox & Snell and Nagelkerke determination pseudo coefficients *R*^2^ were used to explain the general percentage of data variance of the multinomial logistic regression.

Mplus 8.2 was used to run the CFA and measurement invariance analyses. For all other data analyses, including multinomial logistic regression, IBM SPSS Statistics 25 was used.

## Results

### Trauma exposure

More than half of the total sample (61.6%) reported at least one traumatic experience throughout a lifetime. However, significant differences were found in trauma exposure between Lithuanian and Japanese adolescents (see Table 2), with a higher prevalence of trauma exposure in Lithuanian (80.0%) *v*. Japanese sample (44.6%). Adolescents reported a mean of 1.62 (s.d. = 1.99) traumatic events in a total sample. A significantly higher cumulative trauma was found in the Lithuanian (*M* *=* 2.47, s.d. *=* 2.17), as compared to the Japanese sample (*M* *=* 0.85, s.d. *=* 1.44) (*t*(df) = 18.28(1412.69), *p* < 0.001). Moreover, Lithuanian adolescents experienced significantly more multiple traumatic events (see Table 2).

The most prevalent traumatic experiences in the Lithuanian sample were serious accidents or injuries (48.1%), witnessed physical assaults in a community (40.1%) and stressful or scary medical procedures (38.6%). In comparison, in the Japanese sample, natural disasters (17.5%), physical assaults in the family (11.3%) and serious accidents or injuries (11.3%) were among the most prevalent experiences. Adolescents in Japan reported significantly more experiences of natural disasters (17.5%) *v*. 9.9% Lithuanian sample. Lithuanian adolescents were more often exposed to more interpersonal traumatic experiences than the Japanese sample, e.g. physical abuse in a family, witnessing physical abuse in a family, sexual assault or harassment and witnessing a physical attack (see Table 2).

### Structural validity and measurement invariance of the ITQ-CA

The ITQ-CA CFA results of Lithuanian, Japanese and a total sample are presented in Table 3. Model 1 had a poor fit in all samples; thus, it was rejected. Model 3 had a good fit based on most indicators, but low fit on RMSEA in Lithuanian and total samples, RMSEA and TLI in Japanese sample, so model 3 was also rejected. Results showed the best fit for models 2 and 4 in Lithuanian and a total sample and model 2 in a Japanese sample. Due to lower SRMR indices in model 2 compared to model 4 in Lithuanian and total samples, model 2 – a first-order correlated six-factor model – was chosen as having the best fit in the Lithuanian, Japanese and the total study samples.

**Table 3.** Model fit of confirmatory factor analysis models

Model	RMSEA [90% CI]	SRMR	CFI	TLI	*χ*^2^ (df)	*p*
Japanese sample (*N* = 408)						
Model 1	0.137 [0.126–0.149]	0.084	0.769	0.717	468.5 (54)	<0.001
Model 2	0.070 [0.056–0.085]	0.028	0.972	0.953	117.8 (39)	<0.001
Model 3	0.113 [0.101–0.125]	0.062	0.913	0.880	297.7 (48)	<0.001
Model 4	0.085 [0.072–0.098]	0.044	0.952	0.932	185.7 (47)	<0.001
Lithuanian sample (*N* = 666)						
Model 1	0.119 [0.110–0.128]	0.071	0.827	0.788	562.8 (54)	<0.001
Model 2	0.067 [0.056–0.078]	0.030	0.971	0.951	155.5 (39)	<0.001
Model 3	0.091 [0.082–0.101]	0.054	0.934	0.910	314.5 (48)	<0.001
Model 4	0.076 [0.066–0.086]	0.040	0.956	0.938	226.3 (47)	<0.001
Total sample (*N* = 1074)						
Model 1	0.123 [0.116–0.130]	0.075	0.808	0.765	935.1 (54)	<0.001
Model 2	0.066 [0.057–0.074]	0.026	0.973	0.955	219.4 (39)	<0.001
Model 3	0.097 [0.089–0.104]	0.056	0.928	0.901	530.6 (48)	<0.001
Model 4	0.078 [0.071–0.086]	0.040	0.954	0.935	356.7 (47)	<0.001

RMSEA, root-mean-square error of approximation and 90% confidence interval; SRMR, standardised root-mean-square residual; CFI, comparative fit index; TLI, Tucker–Lewis index.

The results of the ITC-CA measurement invariance testing are presented in Table 4. The configural, metric and scalar models of the ITQ-CA demonstrated a good fit. The changes between configural and metric models were ΔCFI = 0.005 and ΔRMSEA = 0.002. The changes between metric and scalar models were ΔCFI = 0.017 and ΔRMSEA = 0.012. Thus, metric invariance was confirmed across both countries' study samples.

**Table 4.** Results of measurement invariance tests by country (*N* = 1074)

	Model fit indices			Model comparisons	
	*χ*^2^ (df)	CFI	RMSEA [90% CI]	ΔCFI	ΔRMSEA
Posttraumatic stress symptoms					
Configural	201.24 (78)	0.974	0.054 [0.045–0.0064]		
Metric	227.98 (84)	0.969	0.056 [0.048–0.065]	0.005	0.002
Scalar	314.54 (90)	0.952	0.068 [0.060–0.076]	0.017	0.012

*χ*^2^, chi-square; df, degrees of freedom; CFI, comparative fit index; RMSEA, root-mean-square error of approximation; CI, confidence interval; Δ, change in the parameter.

### Factors associated with PTSD and CPTSD

PTSD prevalence among the sample of adolescents in Lithuania was 5.2% (95% CI 3.8–6.9%) (*n* = 43), and in Japan, 2.3% (95% CI 1.4–3.5%) (*n* = 21). CPTSD in Lithuanian sample was 12.3% (95% CI 10.1–14.7%) (*n* = 102), and 4.1% (95% CI 2.9–5.5%) (*n* = 37) in Japan. Of adolescents who reported experiencing at least one traumatic event, 6.5% (95% CI 4.7–8.6%) and 5.2% (95% CI 3.2–7.8%) met the diagnostic criteria for PTSD in Lithuania and Japan, respectively. CPTSD prevalence among adolescents with trauma exposure was 15.3% (95% CI 12.7–18.3%) in Lithuanian and 9.1% (95% CI 6.5–12.3%) of Japanese samples. PTSD prevalence among trauma-exposed adolescents was similar in both Lithuania and Japan, *χ*^2^(df) = 0.77(1), *p* = 0.379, whereas CPTSD was more prevalent in the Lithuanian sample, *χ*^2^(df) = 8.76(1), *p* *=* 0.003.

Multinomial logistic regression models' Likelihood Ratio Tests showed good model fit for the total (*χ*^2^(df) = 274.66(12), *p* < 0.001), Japanese (*χ*^2^(df) = 80.32(10), *p* ≤ 0.001) and Lithuanian (*χ*^2^(df) = 209.56(10), *p* ≤ 0.001) adolescents samples. Cox & Snell and Nagelkerke determination pseudo coefficients *R*^2^ explained 22.7–32.4% data variance for the total sample, 17.9–28.2% and 27.2–37.2% in Japanese and Lithuanian samples, respectively.

The country was not a significant predictor in the multinomial logistic regression model in the aggregated total sample; therefore, we conducted logistic analysis in the total sample, as well as for the separate country datasets (see Table 5). In the total sample after controlling for country effect, both PTSD and CPTSD (compared to none trauma-related disorder) were predicted by cumulative trauma (PTSD OR = 1.51, *p* = 0.003; CPTSD OR = 1.83, *p* < 0.001), female gender (PTSD OR = 2.36, *p* = 0.004; CPTSD OR = 2.28, *p* = 0.001), and social factors such as more financial difficulties in family (PTSD OR = 2.91, *p* = 0.018; CPTSD OR = 3.07, *p* = 0.002) and loneliness (PTSD OR = 1.25, *p* = 0.007; CPTSD OR = 1.86, *p* < 0.001). Multinomial logistic analysis in Lithuania and Japan revealed slightly different patterns of significant predictors for PTSD and CPTSD. Cumulative trauma (OR = 1.82, *p* = 0.001) and higher loneliness (OR = 1.24, *p* = 0.037) were significant predictors for PTSD in Lithuanian but not Japanese samples. Financial difficulties (OR = 2.91, *p* = 0.009) were significantly associated with CPTSD risk only in the Japanese sample.

**Table 5.** Multinomial logistic regression for ICD-11 PTSD and CPTSD prediction among adolescents exposed to traumatic experiences (*N* = 1074)

	PTSD *v*. none			CPTSD *v*. none			CPTSD *v*. PTSD		
	Total	Japan	Lithuania	Total	Japan	Lithuania	Total	Japan	Lithuania
			
	OR [95% CI]			OR [95% CI]			OR [95% CI]		
Cumulative trauma	1.51 [1.15, 2.00]**	1.06 [0.60, 1.86]	1.82 [1.30, 2.56]**	1.83 [1.47, 2.28]***	1.68 [1.12, 2.52]**	1.96 [1.50, 2.56]***	1.21 [0.87, 1.66]	1.59 [0.84, 3.02]	1.08 [0.73, 1.58]
Gender (female)	2.36 [1.32, 4.23]**	0.92 [0.35, 2.43]	4.95 [2.00, 12.21]**	2.28 [1.42, 3.67]**	0.76 [0.33, 1.77]	1.96 [1.50, 2.56]***	0.97 [0.48, 1.95]	0.83 [0.25, 2.76]	0.87 [0.29, 2.50]
Loneliness	1.25 [1.06, 1.47]**	1.23 [0.92, 1.64]	1.24 [1.01, 1.52]*	1.86 [1.63, 2.13]***	1.98 [1.53, 2.58]***	1.81 [1.54, 2.12]***	1.49 [1.23, 1.81]***	1.61 [1.12, 2.33]*	1.46 [1.15, 1.84]**
Social support	1.01 [0.96, 1.07]	0.93 [0.84, 1.03]	1.05 [0.98, 1.12]	0.93 [0.89, 0.96]***	0.92 [0.84, 0.99]*	0.93 [0.88, 0.97]**	0.91 [0.86, 0.97]**	0.99 [0.88, 1.01]	0.86 [0.82, 0.96]**
Financial difficulties in family	2.91 [1.02, 7.01]*	2.75 [1.08, 7.01]*	–	3.07 [1.49, 6.33]**	2.91 [1.30, 6.47]**	1.83 [0.31, 10.61]	1.06 [0.38, 2.93]	1.06 [0.34, 3.29]	–
Country (Lithuanian)	1.03 [0.52, 2.01]	–	–	1.09 [0.62, 1.93]	–	–	1.06 [0.47, 2.43]	–	–

OR, odds ratio; CI, confidence interval.

*p < 0.05, **p < 0.01, ***p < 0.001.

Loneliness significantly differentiated CPTSD diagnostic status from PTSD. Higher loneliness in a total sample OR = 1.49, *p* < 0.001, and in both studied country samples was a significant predictor for CPTSD *v*. PTSD (Japan OR = 1.61, *p* = 0.030; Lithuania OR = 1.46, *p* = 0.002). Furthermore, low perceived social support was a significant differentiating factor for CPTSD *v*. PTSD in the Lithuanian sample (OR = 0.86, *p* = 0.002) (see Table 5). Moreover, trauma exposure and gender were not-significant predictors for CPTSD *v*. PTSD in the total and both country samples.

## Discussion

The current study explored trauma exposure and PTSD and CPTSD in adolescents in the general population in Japan and Lithuania. Given the different physical, social and cultural contexts, it is not surprising that adolescents in Japan and Lithuania are differentially exposed to potentially traumatic experiences. First, we found that the majority of adolescents were exposed to traumatic experiences in Lithuania, in line with the previous studies on adolescents and youth (Kazlauskas *et al*., 2020; Truskauskaite-Kuneviciene *et al*., 2020). However, only less than half of the adolescents in Japan reported exposure to traumatic events. Second, the profile of specific traumatic experiences in both countries differed. The most prevalent traumatic experience among adolescents in Japan was a natural disaster. In Lithuania, the most prevalent traumatic experiences were associated with accidents or scary medical procedures, but also experiences associated with interpersonal trauma, such as seeing violence in a community or experiencing physical abuse, were highly prevalent. Third, most Japanese adolescents were exposed to a single trauma, whereas multi-traumatisation was more prevalent in the Lithuanian sample. Looking at the findings of trauma exposure, it looks like adolescents in Lithuania are not that safe in comparison to Japan, and these findings corroborate with a recent study of CPTSD in four Asian countries which found that around half of young adults in Japan experienced childhood adversities (Ho *et al*., 2020). Trauma exposure differences in both countries could be rooted in deeper cultural reasons beyond the scope of our study methodology, but might be associated with child protection legislation and diverse societal development in the studied countries (Lozano *et al*., 2018).

Based on ICD-11, we were able to screen for not only PTSD but also CPTSD in our study and test the structural validity of CPTSD symptoms. The six-factor correlated ICD-11 PTSD and CPTSD model supported in our study had a good fit in multiple previous studies (Redican *et al*., 2021), including adolescent studies (Haselgruber *et al*., 2020a; Kazlauskas *et al*., 2020; Redican *et al*., 2022). Moreover, measurement invariance of PTSD/CPTSD symptom measure was supported in both countries. Overall, the prevalence of PTSD and CPTSD was different in Japan and Lithuania, associated with different trauma exposure levels. However, controlling for trauma exposure in a subsample of adolescents exposed to traumatic experiences, we found a very similar prevalence of PTSD in both country samples, with 6.5 and 5.2% in Lithuania and Japan, respectively, but not CPTSD, which was more prevalent in the Lithuanian sample, 15.3% compared to Japanese adolescents 9.1%. Higher rates of CPTSD in Lithuanian, compared to Japanese adolescents, are in line with a higher prevalence of cumulative trauma (including the rates of interpersonal trauma) among Lithuanian adolescents in the current study. The aforementioned findings also correspond to the previous studies conducted in adolescent populations (Sachser *et al*., 2017b; Elliott *et al*., 2021; Radican *et al*., 2022). However, overall prevalence of PTSD and CPTSD in our study was higher in comparison to a recent study of trauma-exposed adolescents from general population of the Northern Ireland, which reported 3.4% CPTSD and 1.5% PTSD prevalence based on ITQ-CA criteria (Redican *et al.*, 2022).

The findings of the study extend current knowledge regarding the factors associated with PTSD and CPTSD in adolescence. As expected, PTSD and CPTSD were predicted by cumulative trauma exposure; also, the female gender was found to be a risk factor in line with previous studies. However, we also found that social and interpersonal factors in adolescence, such as financial difficulties in families, lower social support and loneliness, are highly relevant for PTSD and CPTSD. In particular, loneliness was a significant factor differentiating PTSD and CPTSD in both countries. These results corroborate previous studies in which higher rates of loneliness were reported in the adult group with a higher risk for CPTSD (Zerach *et al*., 2019). The significant link between loneliness and poorer mental health in children and adolescents was also proved meta-analytically (Loades *et al*., 2020). Moreover, poorer perceived social support was also related to higher rates of the symptoms of CPTSD (Simon *et al*., 2019).

### Strengths and limitations

The cross-sectional design of our study does not reveal trajectories of traumatic stress symptoms following trauma exposure. It is possible that social-interpersonal factors important for PTSD and CPTSD development can be risk factors but also develop as a result or in parallel to CPTSD symptoms, which also include emotional regulation and interpersonal difficulties. Considering we found that interpersonal factors are essential in adolescents exposed to traumatic experiences, the COVID-19 pandemic could have significantly impacted the findings. The prolonged closure of schools, remote teaching and other social restrictions negatively impacted the mental health of adolescents (Loades *et al*., 2020). PTSD and CPTSD symptoms could be elevated by difficulties caused by the COVID-19 pandemic-related stressors. For example, a recent meta-analysis demonstrated that the prevalence of depression and anxiety in children and adolescents during the COVID-19 pandemic was increased (Racine *et al*., 2021).

Furthermore, the COVID-19 pandemic had an impact on our data collection methods as well. Data were collected via an online platform, which could have an impact on the study findings. Moreover, we used different data collection approaches in Lithuania and Japan. In Lithuania, data in an educational setting in schools across the country were collected. In Japan, the survey platform was used via parental subscription. As financial incentives were provided for participation in Japan, it is possible that families with financial difficulties were more motivated to participate in the study, even though the financial reward was small. We used a rigid procedure to synchronise our measures in both countries, and measurement invariance was confirmed in both countries for PTSD/CPTSD measures. However, it is possible that due to the cultural differences, adolescents responded differently to the items in our measures, such as traumatic experiences or symptoms. Further studies are needed to explore cultural factors on traumatic stress symptoms.

## Conclusions

Traumatic stress symptoms are universal across regions and cultures. However, the specific cultural context significantly affects traumatic experiences in each region, and ways of coping with adversities in life are rooted in the culture, healthcare or social care systems. Cross-cultural studies on traumatic stress that use the same measures are very important for the future of child and adolescents mental health research. The current study showed that PTSD and CPTSD symptom structure is similar in very different countries in Asia and Europe, even though the traumatic experiences vary a lot. Moreover, social interpersonal factors, such as loneliness, were even more significant than country factors showing that social support and connectedness with others are crucial for adolescents across the globe. Our study provides insights for further development of treatment for PTSD and CPTSD for adolescents, which should consider addressing the loneliness of traumatised youth.

## Data

The STAR-A data are not currently freely available to researchers in general due to ethical requirements. Interested researchers can directly contact the corresponding author at: evaldas.kazlauskas@fsf.vu.lt
